# Longitudinal changes in amygdala–supplementary motor area connectivity and their association with recurrent self-harm in adolescents with mood disorders

**DOI:** 10.1186/s12888-026-08253-0

**Published:** 2026-06-05

**Authors:** Jiayi Feng, Ran Zhang, Yongxia Ren, Jingshuai Zhou, Junjie Zheng, Yufei Wu, Yuenan Yu, Rongxin Zhu, Fei Wang

**Affiliations:** 1 School of Psychology, Henan Medical University, Xinxiang, 453003 China; 2 Early Intervention Unit, Department of Psychiatry, The Affiliated Brain Hospital of Nanjing Medical University, Nanjing, 210029 China; 3 Functional Brain Imaging Institute of Nanjing Medical University, Nanjing, China; 4 Department of Mental Health, School of Public Health, Nanjing Medical University, Nanjing, China; 5 School of Public Health, Southeast University, Nanjing, China

**Keywords:** Mood disorder, Adolescent, Longitudinal, Neuroimaging, Resting-state, Functional connectivity, Repeated self-harm behavior

## Abstract

**Background:**

Adolescents hospitalized with mood disorders face a heightened risk of repeated self-harm (SH) after discharge. Neuroimaging phenotype may complement traditional symptom-based approaches by revealing neural mechanisms of SH vulnerability. This study aimed to investigate longitudinal changes in functional connectivity (FC) in adolescents with repeated SH and to examine how these neural dynamics relate to SH-related symptoms.

**Methods:**

We recruited 201 adolescent inpatients with mood disorders and SH behaviors, who were classified into repeated (RESH; *n* = 63) and non-repeated (NRESH; *n* = 138) SH groups based on a six-month follow-up. Resting-state fMRI and clinical assessments were conducted at three time points: acute (T1, admission ≤ 1 week), subacute (T2, 1–2 weeks), and discharge (T3). Voxel-wise ANCOVA identified regions showing significant group-by-time interaction effects in amygdala functional connectivity. Partial least squares correlation (PLSC) was used to examine associations between changes in FC (ΔFC) and suicidal symptoms, while logistic regression tested whether baseline and dynamic FC predicted SH recurrence at follow-up.

**Results:**

ANCOVA revealed significant group-by-time interaction effects in amygdala-cortical connectivity, particularly with the left supplementary motor area (L-SMA) (Gaussian random field, GRF corrected, *P* < 0.05). PLSC showed that ΔFC between the amygdala and L-SMA was significantly associated with suicidal measures. Logistic regression indicated that both baseline (AUC = 0.75, 95% CI: 0.63–0.79) and ΔFC (AUC = 0.76, 95% CI: 0.68–0.82) between the amygdala and L-SMA, along with sex and Beck Scale for Suicide Ideation (BSS) item 3(“Reasons for Living or Dying”), were significant predictors of SH behavior.

**Conclusions:**

Longitudinal changes in amygdala-L-SMA connectivity are associated with suicidal symptoms and predict SH recurrence, supporting the integration of neurobiological and clinical indicators for early suicide risk stratification.

**Clinical trial number:**

Not applicable.

**Supplementary Information:**

The online version contains supplementary material available at 10.1186/s12888-026-08253-0.

## Introduction

Psychiatrically hospitalized adolescents are a particularly vulnerable group as they are at high risk for repeated self-harm (SH) behaviors [1, 2]. Approximately one in five patients with SH behaviors experienced repeated SH behaviors after discharge, and one in every two hundred patients is at risk of death [3]. However, traditional SH assessments have shown limited prognostic and predictive value [4–6], highlighting an urgent need for novel predictors of SH. In response, recent work has focused on identifying brain–behavior/symptom associations related to SH, which may not only complement traditional risk assessments but also advance neurobiological understanding and prediction of SH [7–9].

There has been a substantial growth in neuroimaging studies over the past decade examining the brain circuitry underlying SH behavior [10–12]. Growing evidence from neuroimaging studies suggests that individuals with SH behavior exhibit widespread aberrant connectivity across multiple brain networks involved in emotion regulation [13, 14], inhibitory control [15, 16], cognitive flexibility [17, 18], and reward-related processes [19–21]. The amygdala–frontoparietal pathway has been identified as a key region of interest in SH behavior [22–25]. Prior research has demonstrated that individuals engaging in SH behavior exhibit heightened amygdala reactivity to emotional stimuli compared to healthy controls [26, 27]. Furthermore, abnormalities in amygdala circuitry have been observed in adolescents and young adults with SH behavior [28]. As a key brain region involved in emotional reactions, decision-making, and memory processing [26, 29, 30], the amygdala’s functional connectivity alterations may be relevant to SH, and its biological significance in psychiatrically hospitalized adolescents warrants further exploration.

Despite these initial positive findings, given the cross-sectional nature of prior studies, it remained an open question if these risk factors are temporally dynamic or predictive of future SH behavior. Compared to cross-sectional studies, longitudinal investigations can better capture the temporal dynamics of neural changes and their relationship to the progression of SH behaviors. Using longitudinal data from depressed adolescents with a history of SH, we identified individuals who reported repeated SH within six months post-discharge (RESH) and those who did not (NRESH). Then, leveraging neuroimaging (functional connectivity) and clinical symptom data (suicide assessment variable) collected during hospitalization from both groups, we conducted a longitudinal and multivariable design. First, we examined amygdala–whole brain functional connectivity at three time points during hospitalization—acute (≤ 1 week, T1), subacute (1–2 weeks, T2), and remission (discharge, T3)—and found that longitudinal changes in connectivity patterns differed between the two groups. Next, we applied partial least squares correlation (PLSC) analysis to identify RESH group-specific functional connectivity patterns associated with clinical assessment variables. Additionally, we employed logistic regression analysis to further evaluate whether baseline connectivity or its longitudinal changes could predict SH recurrence within six months post-discharge. Collectively, this study aimed to characterize the temporal dynamics of brain function alterations preceding repeated SH episodes, and highlight these change may serve as potential biomarker for future SH risk.

## Method

### Participants

Participants included 201 adolescent inpatients aged 12 to 18 years (Mean = 14.95, SD = 1.43), recruited from a psychiatric inpatient facility in Affiliated Nanjing Brain Hospital, Nanjing Medical University, China. Written informed consent was obtained, and the study was approved by the Ethics Committee of Affiliated Nanjing Brain Hospital, Nanjing Medical University. All participants were evaluated by two professionally trained psychiatrists using the Schedule for Affective Disorders and Schizophrenia for School-age Children-Present and Lifetime Version (K-SADS-PL), which is based on the DSM-IV criteria and assesses both current and past psychiatric episodes in children and adolescents. All patients received standardized pharmacological treatment consistent with clinical practice guidelines [31], with detailed information presented in Table 1. Inclusion criteria required participants to meet the criteria for a primary diagnosis of mood disorders or dysthymia and a history of SH behavior. Exclusion criteria included: (1) MRI contraindications; (2) History of head trauma with loss of consciousness ≥ 5 min; (3) Diagnosed neurological disorders; (4) Major medical conditions potentially affecting brain tissue; (5) Current substance use disorders (alcohol or drug abuse/dependence). All patients were maintained on a stable psychotropic medication regimen throughout hospitalization.

**Table 1:** Demographic and clinical characteristics of study participants (*N* = 201)

Variables	RESH(*n* = 63) mean ± SD	NRESH(*n* = 138) mean ± SD	T/X^2^	*p*-value
Age(year)	14.78 ± 1.48	15.06 ± 1.40	1.28	0.20
Female(%)	58(92.1)	100(72.5)	9.88	0.002**
Diagnosis(MDD/BD)	28/35	74/64	1.45	0.23
Education(year)	9.27 ± 1.91	9.15 ± 2.03	0.39	0.70
Medication(yes)				
Mood stabilizers (%)	63(100)	138(100)	/	/
Antidepressants (%)	24(38.10)	55(39.86)	0.06	0.81
Antipsychotics (%)	63(100)	138(100)	/	/
Anxiolytics (%)	15(23.80)	23(16.67)	1.43	0.23
Sedatives (%)	53(84.12)	104(75.36)	1.94	0.16
T1				
HAMD-17	25.37 ± 5.31	23.95 ± 6.21	1.53	0.13
HAMA	24.73 ± 6.60	23.21 ± 6.99	1.42	0.16
BSS	43.18 ± 27.04	27.53 ± 24.47	3.86	< 0.001***
T2				
HAMD-17	18.70(5.74)	17.09(6.47)	1.68	0.09
HAMA	18.60(6.08)	17.34(7.01)	1.24	0.22
BSS	26.08(25.08)	16.40(19.78)	2.68	0.008**
T3				
HAMD-17	9.73 ± 5.79	8.31 ± 4.78	1.82	0.07
HAMA	10.65 ± 6.26	9.55 ± 6.26	1.15	0.25
BSS	8.45 ± 14.16	6.85 ± 14.24	0.64	0.52

Abbreviation. SD, Standard Deviation; Repeated Episodes of Self-Harm group (RESH); Non-Repeated Episodes of Self-Harm group (NRESH); HAMD_17, 17-items Hamilton Depression Rating Scale; HAMA, Hamilton Anxiety Rating Scale; BSS, Beck Scale for Suicidal Ideation; MDD, major depressive disorder; BD, bipolar disorder; T1, baseline; T2, 1–2 weeks; T3, discharge

*P < 0.05, **P < 0.01, ***P < 0.001

### Suicide assessment

All patients initially were assessed with MRI data, SH behavior and clinical symptoms during hospitalization (T1: MRI data, SH behavior and clinical symptoms) immediately following consent, typically within 2 to 4 days of admission. Post-baseline assessments were completed at 1 week (T2: MRI data, SH behavior and clinical symptoms), at discharge (T3: MRI data, SH behavior and clinical symptoms) and at 6 months post-discharge (T4: SH behavior).

SH behavior including suicide attempts (SA) and non-suicidal self-injury (NSSI) was assessed through medical history and physician interviews conducted by trained doctors. SA refers to a SH act with at least one recorded instance of intent to die. NSSI is defined as deliberate SH without lethal intent, occurring five or more times within a year. The specific interview methods and steps included: “Did you try to hurt yourself and how?”, “Have you ever cut, burn, or hit (etc.) yourself?”, “How many times have you done this kind of behavior?” Suicidal and non-suicidal adolescents were recruited to ensure variability in the constructs of interest for this investigation. Symptom severity was measured using the Beck Scale for Suicidal Ideation (BSS) [32], a 19-item self-report scale. The BSS is extracted into three factors: active suicidal desire (1, 2, 3, 4, 6, 7, 8, 9, 10, 11, 15,18), preparation (12, 13, 16), and passive suicidal desire (5, 14, 19) [33]. The depression and anxiety symptoms were assessed using the 17-item Hamilton Depression Rating Scale (HAMD-17) [34]and the Hamilton Anxiety Rating Scale (HAMA) [35].

Based on results of the suicide assessment at the six-month follow-up period after patient discharge, participants were exclusively classified as: (a) repeated episodes of self-harm (RESH; *n* = 63) if there was current suicidal ideation and a report of a self-harm behavior in the follow-up period (T4) or (b) no-repeated episodes of self-harm (NRESH; *n* = 138) if there was current suicidal ideation with no self-harm behavior(T4). Among the 201 participants, 125 (63%) already had a history of repeated self-harm, while 76 (37%) presented with a first episode. Importantly, we note that a history of repeated self-harm was more prevalent at baseline in the group that went on to have recurrent episodes (RESH group: 48/63, or 76%) compared to the non-recurrent group (NRESH group: 77/138, or 55%).

### MRI data acquisition

We acquired functional and structural T1-weighted MRI brain scans at the Nanjing Brain Hospital of Nanjing Medical University using a Siemens MAGNETOM Prisma 3.0T MRI scanner (syngo.via software platform) with a 64-channel neurofunctional coil. MRI data collection parameters were as follows:

Resting-state functional imaging (rs-fMRI): repetition time 500 ms; echo time 30 ms; field of view (FOV) 224 mm; voxel size 3.5 mm^3^ isotropic; slice thickness/gap of 3.5 mm/0.5 mm, a total of 35 slices; acquisition time 8.07 min. High-resolution structural imaging (sMRI): repetition time 2530 ms; echo time 2.98 ms; flip angle = 7°; FOV = 256 × 224 mm², voxel size = 0.5 × 0.5 × 1 mm³, slice thickness = 1 mm, 192 slices; acquisition time 5.58 min.

### MRI preprocessing and data extraction

Functional MRIs underwent preprocessing using the well validated tools Statistical Parametric Mapping 12 (SPM12; http://www.fil.ion.ucl.ac.uk/spm).and Data Processing Assistant for R-fMRI (DPARSF; http://www.restfmri.net/forum/) [36]. The first 10 time points were discarded due to the instability caused by initial magnetic field fluctuations. The remaining images were corrected for slice timing and head motion with the reference plane being the 1st slice. Subjects with excessive motion (> 3 mm displacement and/or 3◦ degrees of motion) were excluded. The images also underwent the following preprocessing steps: normalization to the standard Montreal Neurological Institute (MNI) space using the Diffeomorphic Anatomical Registration Through Exponentiated Lie Algebra (DARTEL) tool, followed by resampling to a 3 × 3 × 3 mm resolution. To reduce physiological noise, nuisance covariates were regressed using the Friston-24 model, including white matter and cerebrospinal fluid signals, as well as head motion parameters. Additionally, a linear detrend was applied to reduce BOLD signal drift.

Seed-based resting-state functional connectivity (rs-FC) analysis was conducted via DPABI to examine brain connectivity. The amygdala was selected as the seed region of the rs-FC analysis, defined using the automated anatomical labeling (AAL) template from the DPABI toolkit (http://www.restfmri.net) [37], resampled to a voxel size of 3 × 3 × 3 mm. First, after preprocessing, the data underwent band-pass filtering (0.01–0.08 Hz) and spatial smoothing with a 6-mm FWHM Gaussian kernel. Second, FC maps were then generated by computing linear correlations between the average time series within the ROI and all brain voxels. Finally, Fisher’s r-to-z transformation was applied to create subject-specific maps for further statistical analysis.

### Statistical analyses

Descriptive statistics were first conducted to examine group differences in sociodemographic, medication use and baseline variables using independent samples t-tests and χ^2^ test.

Second, SPM12 was used to examine longitudinal group differences in brain connectivity based on FC z maps to assess interaction effect. An analysis of covariance (ANCOVA) model was conducted with one within-subject factor (time point: T1, T2, T3) to assess group-by-time interaction effects on amygdala–whole brain connectivity between patients with RESH and NRESH groups, controlling for age and sex. FC z-maps were computed to evaluate the main effects of group, time, and their interactions (i.e., group × time), followed up by a Gaussian random field (GRF) correction with a significance threshold of *P*_GRF_ < 0.05 (cluster size ≥ 50 voxels). Furthermore, to account for the potential impact of pharmacological treatment during hospitalization, a sensitivity analysis was subsequently performed. This additional ANCOVA model included medication type (i.e.,atypical antipsychotics, mood stabilizers, antidepressants, anxiolytics, and sedative-hypnotics) as binary covariates (c coded as 1 if the medication class was administered during hospitalization and 0 if not), in order to evaluate the robustness of the observed group-by-time interaction effects.

Third, we employed PLSC analysis to examine the multivariate associations within the RESH group between T3-T1 longitudinal changes (Δ) in brain and suicidal measures, relating brain variables (extracted FC value from regions exhibiting significant group-by-time FC interactions) to 19 suicide-related variables (BSS scores) [38]. Because our study primarily focused on self-harm, and baseline analyses showed that BSS, rather than HAMD or HAMA, significantly differentiated the RESH and NRESH groups, we did not include HAMD or HAMA in subsequent analyses. In PLSC, each variable was centered (having a mean of 0) and normalized (the sum of squared values equal to 1). PLSC allowed us to identify significant latent variable (LV) pairs that maximized the covariance between the two data matrices: X (FC features) and Y (suicide-related measures). Each LV pair consisted of one component from the brain variables (FC scores) and one from the symptom variables (suicide scores), together capturing the maximal shared variance between the two domains. The significance of each LV was assessed using permutation tests, while the stability of the loadings for each contributing variable was evaluated through bootstrap resampling. Both permutation and bootstrap procedures were conducted with 1,000 iterations, and variable stability was considered reliable at a bootstrap threshold of |z| > 1.96 as well as 10-fold cross-validation. All data analyses were performed using R 4.1.2 with PLSC being performed using the *TExPosition* and the *data4PCCAR* packages (https://github.com/HerveAbdi/data4PCCAR).

Finally, to test whether the baseline and changed features (brain and suicide) could predict SH behaviors at the 6-month follow-up, we conducted multiple logistic regression analyses, separately. Each model included sex, age, corresponding baseline/changed (Δ) regional FC value and clinical variables to evaluate whether the found features could predict follow-up outcomes independently of these risk measures. We report standardized odds ratios as well as area under the curve from receiver operating characteristic curves to assess the accuracy of the models in predicting behavior onsets. To reduce potential overfitting and provide internally validated estimates of model performance, propensity score weighting (PSW), class-weighted logistic regression and nested cross-validation was applied to the logistic regression models.

## Result

### Demographics and clinical characteristics

Within six months post-discharge(T4), 63 participants (31%) of the total 201 participants engaged in the RESH group. No significant differences were found between the RESH and NRESH groups in age, education, HAMD-17, or HAMA scores at baseline (T1). However, significant group differences emerged in sex, and the RESH group showed significantly higher BSS scores at baseline (T1) compared to the NRESH group (*P* < 0.001, Table 1). Additionally, there were no significant differences in either medication tyoes or the average dosages administered timing between the two groups during hospitalization (*P* >0.05, see Table 1 and Supplementary Table S5).

### Longitudinal differentiation of functional connectivity across RESH and NRESH group

We were interested in investigating the differences in amygdala-to-whole-brain connectivity between the RESH and NRESH group, as well as how this connectivity pattern may change across their longitudinal recovery processes. Therefore, we primarily focus on ANCOVA analysis between the two groups. Detailed results of the cross-sectional comparisons between the two groups are provided in the Supplementary Materials (see Supplementary Table S1).

Significant group effects (RESH > NRESH) were observed in FC between the amygdala and multiple brain regions, including the right paraHippocampal (R-PHG), right Inferior frontal gyrus, orbital part (R-IFGoperc), right middle frontal gyrus, orbital part (R-ORBmid), left lingual (L-LING), left calcarine (L-CAL), right superior temporal gyrus (R-STG), left superior parietal lobule (L-SPG), and left supplementary motor area (L-SMA) (GRF corrected, cluster significance *P* < 0.05, voxel significance *P* < 0.05, cluster size ≥ 50; see Supplementary Table S2). In addition, significant time effects were identified in FC between the amygdala and the left and right middle frontal gyri, orbital part (L-ORBmid and R-ORBmid), right superior temporal gyrus (R-STG), right calcarine cortex (R-CAL), left middle temporal gyrus (L-MTG), and left superior occipital gyrus (L-SOG) across baseline and discharge time points in depressed adolescents (GRF corrected, cluster-level significance *P* < 0.05, voxel-level significance *P* < 0.05, cluster size ≥ 50; see Supplementary Table S2).

**Fig. 1:**
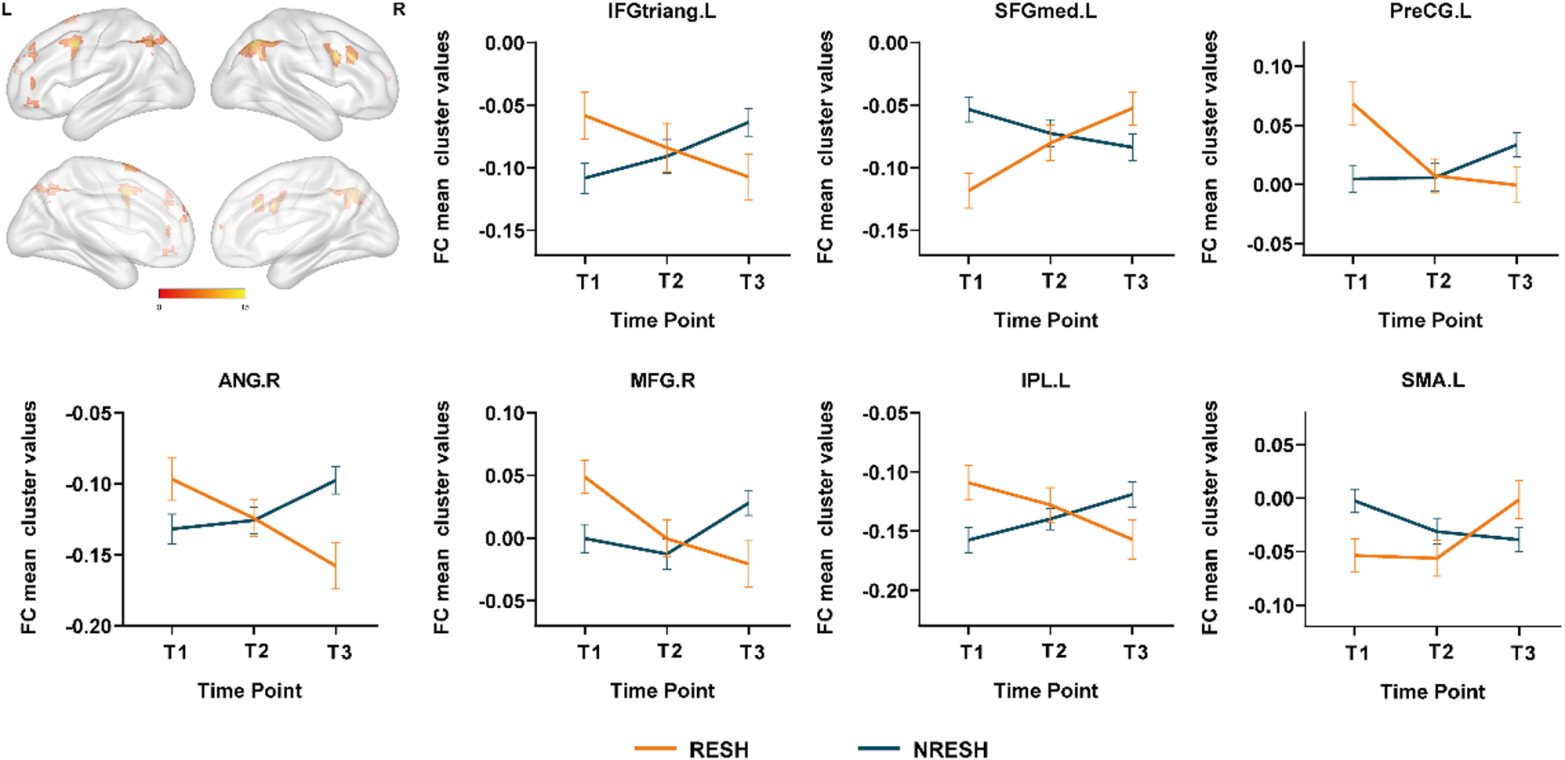
Longitudinal changes in amygdala-based functional connectivity (FC) between RESH and NRESH. Brain maps showing significant group-by-time interactions in FC between the amygdala and key regions (GRF corrected, voxel significance p < 0.05, cluster size ≥ 50).Line graphs depict longitudinal FC changes in seven regions: left inferior frontal gyrus triangular part (L-IFGtriang), left medial superior frontal gyrus (L-SFGmed), left precentral gyrus (L-PrCG), right angular gyrus (R-ANG), right middle frontal gyrus (R-MFG), and left inferior parietal lobule (L-IPL), left supplementary motor area (L-SMA). The yellow lines represent the RESH group, and the blue lines represent the NRESH group. Error bars indicate standard error of the mean (SEM)

Importantly, ANCOVA model revealed a significant group-by-time interaction in the FC between the amygdala and the left Inferior frontal gyrus (L-IFG), left precentral gyrus (L-PrCG), right angular gyrus (R-ANG), right middle frontal gyrus (R-MFG), and left inferior parietal lobule (L-IPL), as well as the left medial superior frontal gyrus, (L-SFG) and left supplementary motor area (L-SMA) (GRF corrected, cluster significance *P* < 0.05, voxel significance *P* < 0.05, cluster size ≥ 50; Table S2). The majority of regions displayed negative interactions, for example, in the FC between the amygdala and the L-IFG, L-PrCG, R-ANG, R-MFG, and L-IPL. Fewer regions, including the L-SFG and L-SMA, exhibited positive interaction effects (Fig. 1). Additionally, all seven voxel-wise clusters differentiating the RESH and NRESH groups remained significant in a series of sensitivity analyses controlling for psychotropic medication use (GRF corrected, cluster significance *P* < 0.05, voxel significance *P* < 0.05, cluster size ≥ 50; see Supplementary Table S3).

### Multivariate relationships of brain-suicide PLSC within RESH group

PLSC analyses were performed to identify multivariable relationships between (changes in) modulatory effects of FC values on all 7 connections-of-interest (extracted FC value from regions exhibiting significant group-by-time FC interactions) and individual suicidal measures in BSS scores. PLSC identified one significant LV (Permutation test 1,000, *P* < 0.05), explaining 73.90% of the brain-suicide covariables.

**Fig. 2:**
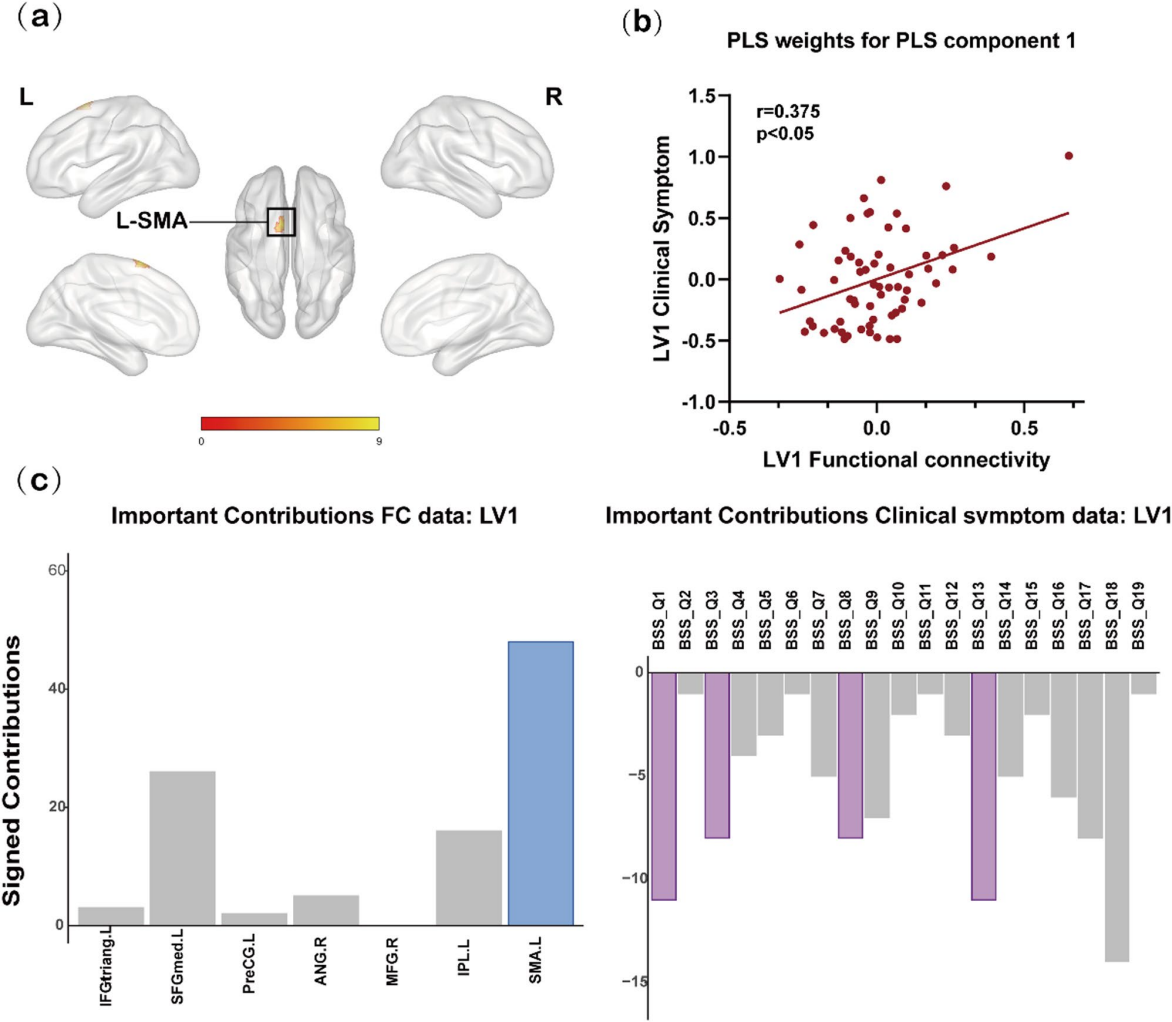
Partial least squares correlation (PLSC) analysis of functional connectivity (FC) and clinical symptoms in RESH. (**a**) The left supplementary motor area (L-SMA); (**b**) A scatterplot of PLS1 scores (a weighted sum of changes in BSS scores) and changes in FC (Pearson’s r = 0.375, p < 0.05); (**c**) Beck Scale for Suicidal Ideation (BSS) items 1, 3, 8(active suicidal desire), 13 (preparation) contribute a significant and stable amount of explained variance to latent variable 1 (LV1). Bootstrap ratios greater than 1.96 are shown with purple; L-SMA contribute a significant and stable amount of explained variance to latent variable 1 (LV1); Bootstrap ratios greater than 1.96 are shown with blue

Overall, the primary PLSC analysis, based on the seven regions showing significant interaction effects, identified a multivariable brain–suicide association in the first latent variable (LV1). Bootstrap resampling (1,000 iterations, |Z| > 1.96) indicated that amygdala–L-SMA connectivity, together with BSS items 1 (Wish to Live), 3 (Reasons for Living or Dying), 8 (Attitude toward Ideation), and 13 (Capability), contributed stably to this latent pattern (Fig. 2C). Collectively, these multivariate associations highlight the role of the L-SMA in representing active suicidal desire and preparatory processes within the RESH group.

In addition, an exploratory PLSC analysis including all brain regions was conducted as a sensitivity analysis. The results were largely consistent with those of the primary analysis (see Supplementary Figure S2).

### Logistic regression models predicting the onset of self-harm behavior at six months post-discharge

To identify predictors of repeated SH behavior at six-month follow-up (T4), we employed a logistic regression model incorporating brain functional connectivity and clinical measures. FC values between the amygdala and the L-SMA, BSS item 3 (Reasons for Living or Dying), and sex were independently associated with SH behavior at the 6-month follow-up (see Supplementary Table S4). Notably, baseline FC between the amygdala and L-SMA, together with sex, significantly predicted SH occurrence (AUC = 0.75, 95% CI: 0.63–0.79; sensitivity: 67%; specificity: 75%; see Supplementary Figure S1). Furthermore, incorporating dynamic FC changes in the amygdala–SMA circuit (ΔFC = T3–T1), along with sex and BSS item 3, improved predictive performance (AUC = 0.76, 95% CI: 0.68–0.82; sensitivity: 65%; specificity: 82%; see Supplementary Figure S1). These findings underscore the potential of combining neuroimaging and clinical variables for early identification of adolescents at risk for SH behavior. The PSW and class-weighted models yielded results similar to the primary logistic regression (see Supplementary Table S6–7). Nested cross-validation showed stable but slightly reduced performance for both models (baseline model: AUC = 69.4%; longitudinal model: AUC = 70.5%, see Supplementary Figure S3). In addition, we compared the baseline-only model with the model including longitudinal FC changes using DeLong’s test, which revealed no significant difference (Z = − 0.82, *P* = 0.41).

## Discussion

To our best knowledge, our longitudinal fMRI study is the first to characterized whether amygdala–whole brain connectivity during the course of inpatient recovery differs between adolescents with and without repeated SH episodes, stratified by their behavior within six months after discharge. We identified seven regions where FC differed in the RESH group and uncovered their dynamic changes over time, while NRESH showed converse trajectories, including L-SFG, L-SMA, L-IFG, L-PrCG, R-ANG, R-MFG, and L-IPL. Using the identified pattern of functional brain alterations, we conducted a multivariate analysis to examine multivariable brain–suicide associations. The strongest neural correlate was an association between active suicidal thoughts (BSS Item 1, 3, and 8), suicide preparation (BSS items 13) and FC between the amygdala and L-SMA. Additionally, in the baseline logistic regression model, a blunted FC between the amygdala and L-SMA was associated with significantly higher odds of engaging in SH behavior (B = -3.93, OR = 0.02). In the longitudinal model, greater changes in FC from acute to remission phrase was positively associated with behavioral outcomes (B = 3.27, OR = 26.30), suggesting that individuals who exhibited greater neural reorganization were more likely to engage in SH behavior. Meanwhile, we found that female patients are more likely to engage in repeated self-harm behaviors (B = 1.57, OR = 4.79). These findings may reflect brain function instability or atypical developmental trajectories, potentially charactering and predicting future repeated SH behavior.

Our voxel-wise analyses identified multiple regions in amygdala-whole brain functional connectivity showing significant group-by-time interaction effects, indicating that adolescents with and without post-discharge self-harm recurrence exhibited distinct trajectories of neural change during hospitalization. Among these, regions within the amygdala-frontoparietal system, including the SMA and PrCG, may be particularly relevant to SH [23, 39, 40]. The PrCG is not only involved in voluntary motor control but also contributes to the integration of emotional and cognitive processes [41]. Previous studies have reported reduced cortical thickness in the PrCG in adolescents with suicidal ideation [42], as well as decreased amygdala–PrCG and amygdala–IPL connectivity [43]. In addition, abnormal connectivity between the PrCG and MFG has been observed in patients with suicide attempts [44]. These findings support the involvement of disrupted interactions across emotional, cognitive, and motor systems in SH behaviors, consistent with our results. On the other hand, some studies have reported an opposite pattern, where reductions in SH frequency were associated with decreased amygdala–SMA connectivity [45], highlighting the need for further replication.

Additionally, multivariable brain–suicide associations between amygdala–L-SMA connectivity and measures of active suicidal thoughts and preparations were observed, which may be relevant to repeated self-harm behavior. A previous study found that deficits in amygdala-frontal connectivity may be associated with depressive symptoms, whereas hyperconnectivity between the amygdala and SMA showed higher specificity in patients with SH behaviors [46]. Consequently, the future risk of repeated SH may be driven by both situational and neuroimaging characteristics, such as the nature of suicidal thinking and brain connectivity in motor control regions like the L-SMA.

Toward this end, we derived both static (baseline) and dynamic (longitudinal change) functional connectivity metrics, which were used as predictors in logistic regression models respectively to distinguish adolescents with and without repeated SH behavior following discharge. Notably, our logistic regression results potentially indicate both stable and temporally dynamic risk factors of future repeated SH behavior, which corresponds to a conceptual model of temporal dynamics in suicide risk [47, 48]. For example, the Fluid Vulnerability Theory (FVT) posits that baseline risk and acute risk interact dynamically, such that individuals with higher vulnerability (i.e., elevated baseline risk) are more susceptible to the activation of suicidal modes when triggered, compared to those with lower vulnerability [48]. Consistent with this framework, our study found that both cross-sectional and longitudinal logistic models showed that baseline functional connectivity (AUC = 0.75, 95%CI: 0.63, 0.79) and its changes from the acute to discharge phase (AUC = 0.76, 95%CI: 0.68,0.82) were significantly associated with behavioral outcomes, underscoring the importance of tracking brain connectivity changes over time, particularly in L-SMA.These finding suggest that blunted and unstable (greater changes) brain function may serve as a marker of future SH risk, where the trajectory of brain connectivity changes may offer a more sensitive marker for identifying adolescents at elevated risk of repeated SH. Overall, compared with most machine-learning studies [49, 50], our model achieved only moderate performance, as the primary goal was to identify key neural correlates rather than to develop a fully validated clinical tool.

While this study offers novel insights, several limitations warrant consideration. First, the modest sample size may limit statistical power and generalizability. Second, external validation of the logistic regression model was not performed, and baseline history of repeated self-harm was not included, which may affect model stability. Third, although neuroimaging features showed potential for predicting short-term recurrence, heterogeneity in self-harm behaviors, particularly the lack of distinction between suicidal and non-suicidal self-injury, may limit the specificity of the findings. Therefore, multimodal approaches that integrate neural, behavioral, and clinical data are likely to achieve higher predictive accuracy and greater clinical utility than single-modality models. In this context, machine learning techniques are particularly advantageous for handling high-dimensional, multimodal datasets and may improve both prediction performance and generalizability. Future studies with larger, multi-center cohorts and multimodal approaches are needed to further examine these findings and to better understand the association between amygdala–SMA connectivity and repeated self-harm behaviors. Additionally, extending follow-up duration, incorporating dynamic clinical measures, and differentiating between subtypes of self-harm will be essential for identifying more specific neurobehavioral pathways.

In conclusion, by integrating multivariate brain–behavior analysis with a longitudinal framework, our findings suggest that changes in amygdala–L-SMA functional connectivity are associated with repeated self-harm behaviors in adolescents with mood disorders. Both baseline connectivity and longitudinal changes were related to post-discharge self-harm recurrence. These findings highlight the potential relevance of dynamic neural measures for identifying youth at suicide risk.

## Supplementary Information

Below is the link to the electronic supplementary material.

Supplementary Material 1

## Data Availability

The data that support the findings of this study are available from the corresponding author upon reasonable request.
