# Supplementary material for: Longitudinal changes in amygdala–supplementary motor area connectivity and their association with recurrent self-harm in adolescents with mood disorders
Source: BMC Psychiatry. 2026 Jun 5;26:617. doi: 10.1186/s12888-026-08253-0 (PMC13471583; doi:10.1186/s12888-026-08253-0)
Supplement: Supplementary file 1 [file 12888_2026_8253_MOESM1_ESM.docx]

**Table S1** Baseline functional connectivity differences between RESH and NRESH

| **Region** | **Voxel** | **MNI coordinates** | | | **F Values^a^** |
| --- | --- | --- | --- | --- | --- |
|  |  | ***X*** | ***Y*** | ***Z*** |  |
| leftt superior temporal gyrus | 533 | -27 | 42 | 48 | -3.82 |
| left calcarine | 454 | 0 | -93 | 3 | 3.68 |
| left paraHippocampal | 57 | -24 | -36 | -6 | 3.40 |
| left middle temporal gyrus | 54 | -51 | -21 | 0 | 3.00 |
| left Inferior frontal gyrus | 70 | -36 | 33 | 9 | 3.24 |
| left superior temporal gyrus | 55 | -54 | -42 | 15 | 3.02 |
| right supraMarginal | 58 | 57 | -33 | 30 | -2.66 |
| right precentral gyrus | 70 | 48 | 0 | 39 | 2.97 |
| left precentral gyrus | 328 | -36 | 3 | 27 | 3.80 |
| right angular gyrus | 111 | 33 | -81 | 48 | 3.48 |
| left inferior parietal lobule | 199 | -33 | -66 | 51 | 3.17 |
| right supplementary motor area | 695 | 6 | -18 | 78 | -3.41 |

^a^ Statistical significance was set at p < 0.05, corrected using Gaussian random field (GRF) correction method. FC, functional connectivities; 𝑋, 𝑌, and 𝑍 coordinates of primary peak locations in the Montreal Neurological Institute space

**Table S2** Brain regions showing significant differences in FC between RESH and NRESH

| **Region** | **Voxel** | **MNI coordinates** | | | **F Values^a^** |
| --- | --- | --- | --- | --- | --- |
|  |  | ***X*** | ***Y*** | ***Z*** |  |
| **Interaction effect** |  |  |  |  |  |
| left Inferior frontal gyrus | 65 | -36 | 39 | 12 | 8.30 |
| left precentral gyrus | 256 | -21 | 45 | 48 | 11.53 |
| left medial superior frontal gyrus | 151 | -45 | 3 | 42 | 14.85 |
| right angular gyrus | 526 | 39 | -48 | 30 | 15.86 |
| right middle frontal gyrus | 210 | 39 | 6 | 27 | 15.69 |
| left inferior parietal lobule | 278 | -33 | -63 | 51 | 14.08 |
| left supplementary motor area | 91 | -6 | 15 | 66 | 9.02 |
| **Main effect of group** |  |  |  |  |  |
| right paraHippocampal | 56 | 15 | -30 | -12 | 15.32 |
| right Inferior frontal gyrus, orbital part | 59 | 27 | 30 | -18 | 12.59 |
| right middle frontal gyrus, orbital part | 50 | 3 | 66 | -15 | 9.48 |
| left lingual | 74 | -9 | -81 | -6 | 8.96 |
| left calcarine | 50 | 3 | -93 | 12 | 9.16 |
| right superior temporal gyrus | 85 | 51 | -33 | 12 | 10.47 |
| left superior parietal lobule | 87 | -21 | -63 | 45 | 10.09 |
| left supplementary motor area | 76 | 6 | -18 | 72 | 10.27 |
| **Main effect of time** |  |  |  |  |  |
| left middle frontal gyrus, orbital part | 173 | -24 | 60 | -6 | 14.68 |
| the right middle frontal gyrus, orbital part | 124 | 24 | 63 | -9 | 17.40 |
| right superior temporal gyrus | 186 | 45 | -12 | -6 | 13.52 |
| right calcarine | 153 | 9 | -63 | 3 | 9.26 |
| left middle temporal gyrus | 142 | -54 | -51 | 12 | 12.78 |
| left superior occipital gyrus | 71 | -15 | -84 | 36 | 10.69 |

^a^ Statistical significance was set at p < 0.05, corrected using Gaussian random field (GRF) correction method. FC, functional connectivities; 𝑋, 𝑌, and 𝑍 coordinates of primary peak locations in the Montreal Neurological Institute space

**Table S3** Sensitivity analyses controlling for psychotropic medication use during hospitalization

| **Region** | **Voxel** | **MNI coordinates** | | | **F Values^a^** |
| --- | --- | --- | --- | --- | --- |
|  |  | ***X*** | ***Y*** | ***Z*** |  |
| **Interaction effect** |  |  |  |  |  |
| left Inferior frontal gyrus | 65 | -36 | 39 | 12 | 8.38 |
| left precentral gyrus | 257 | -21 | 45 | 48 | 11.62 |
| left medial superior frontal gyrus | 154 | -45 | 3 | 42 | 14.82 |
| right angular gyrus | 537 | 39 | -48 | 30 | 15.94 |
| right middle frontal gyrus | 215 | 39 | 6 | 27 | 16.00 |
| left inferior parietal lobule | 286 | -33 | -63 | 51 | 14.31 |
| left supplementary motor area | 94 | -6 | 9 | 66 | 9.15 |

^a^ Statistical significance was set at p < 0.05, corrected using Gaussian random field (GRF) correction method. FC, functional connectivities; 𝑋, 𝑌, and 𝑍 coordinates of primary peak locations in the Montreal Neurological Institute space

**Table S4** Variables independently associated with RESH in the logistic regression models

| **Variables** | **Bsaeline** | | **Change(T3-T1)** | |
| --- | --- | --- | --- | --- |
|  | OR (95%CI) | *P* | OR (95%CI) | *P* |
| Sex | 4.79(1.55,14.78) | ＜0.01** | 3.33(1.03,10.74) | 0.04* |
| Age | 0.83(0.65, 1.06) | 0.13 | 0.77(0.60, 1.00) | 0.05 |
| SMA.L | 0.02(0.00,0.31) | ＜0.01** | 26.30(2.57,269.03) | ＜0.01** |
| BSS_Q1 | 0.77(0.40, 1.50) | 0.45 | 1.36(0.69, 2.69) | 0.38 |
| BSS_Q3 | 1.92(0.98, 3.77) | 0.06 | 2.48(1.24,4.96) | ＜0.01** |
| BSS_Q8 | 1.38(0.80, 2.38) | 0.25 | 0.80(0.52, 1.22) | 0.29 |
| BSS_Q13 | 0.99(0.62, 1.61) | 0.98 | 1.25(0.76, 2.04) | 0.38 |

*OR* adjusted odds ratio of the multivariable binary logistic regression, which set repeated self-harm(Yes vs. No) as the dependent variable, the FC value and clinical variables (age and sex) as the independent variables, *95%CI* 95% confidence interval,*SMA.L*, left supplementary motor area; BSS_Q1, Beck Scale for Suicidal Ideation items 1; BSS_Q3, Beck Scale for Suicidal Ideation items 3; BSS_Q8， Beck Scale for Suicidal Ideation items 8; BSS_Q13， Beck Scale for Suicidal Ideation items 13

*P < 0.05, **P < 0.01, ***P < 0.001

**Table S5** Medication information

|  | **RESH**  **（n=63）** | **NRESH**  **（n=138）** | **T** | **P** |
| --- | --- | --- | --- | --- |
| **Mood stabilizer** |  |  |  |  |
| Lithium (n, mg/d) | 60(920) | 129(940) | -0.52 | 0.60 ^a^ |
| Valproate (n, mg/d） | 7(930.4) | 31(948.6) | -0.17 | 0.86 ^a^ |
| Lamotrigine (n, mg/d) | 32(116.8) | 60(112.1) | 0.16 | 0.87 ^a^ |
| Oxcarbazepine (n, mg/d) | 8(855.6) | 4(704.4) | 0.76 | 0.46 ^a^ |
| **Antipsychotic** |  |  |  |  |
| Quetiapine (n, mg/d) | 59(459.7) | 126(445.1) | 0.60 | 0.54 ^a^ |
| Olanzapine (n, mg/d) | 4(5.5) | 12(8.9) | -0.86 | 0.40 ^a^ |
| Aripiprazole (n, mg/d) | 14(7.8) | 16(11.4) | -1.47 | 0.15 ^a^ |
| **Antidepressant** |  |  |  |  |
| Sertraline (n, mg/d) | 18(61.6) | 42(70.8) | -0.10 | 0.28 ^a^ |
| Fluoxetine (n, mg/d) | 1(20.0) | 2(17.5) | / | / |
| Fluvoxamine (n, mg/d) | 3(83.3) | 4(75) | 0.20 | 0.84 ^a^ |
| escitalopram (n, mg/d) | 1(11) | 3(14.6) | / | / |
| **Anxiolytics** |  |  |  |  |
| Buspirone (n, mg/d) | 15(40.0) | 21(34.1) | 1.29 | 0.20 ^a^ |
| Tandospirone (n, mg/d) | / | 2(17.5) | / | / |
| **Benzodiazepines and related hypnotics** |  |  |  |  |
| Zopiclone (n, mg/d) | 8(7.4) | 13(7.0) | 0.79 | 0.43 ^a^ |
| Lorazepam (n, mg/d) | 46(1.4) | 89(1.6) | -1.37 | 0.17 ^a^ |
| Clonazepam (n, mg/d) | 8(1.8) | 13(1.7) | 0.47 | 0.64 ^a^ |
| Alprazolam (n, mg/d) | 23(0.5) | 37(0.5) | 0.71 | 0.47 ^a^ |

a: by independent-sample t test

**Validated Analysis**

To further evaluate the robustness of our findings, we applied the propensity score weighting (PSW) analysis using the ‘TWANG’ package in R. Logistic regression was first used to estimate the probability of individual exhibiting a given level of amygdala–SMA connectivity, conditional on age, sex, and Beck Scale for Suicide Ideation (BSS) scores. The derived inverse probability of treatment weights (IPTW) was then applied in a weighted logistic regression to reassess the association with recurrent self-harm. At baseline, SMA showed a significant association (OR = 0.03, 95% CI: 0.002, 0.302), with AUC = 0.747 (sensitivity = 0.68, specificity = 0.72). Longitudinally, the association remained significant (OR = 18.58, 95% CI: 1.53 – 225.24), with AUC = 0.76 (sensitivity = 0.63, specificity = 0.86) (Table S10). The PSW analysis confirmed that the associations between amygdala–SMA connectivity and recurrent self-harm remained significant after balancing key covariates (age, sex, and BSS scores), with results consistent with our original logistic regression. This suggests that the observed brain–behavior associations are not merely due to sample imbalance or inflated observed effects, thereby supporting the robustness of our findings.

To address the class imbalance between the RESH group (n = 63) and the NRESH group (n = 138), we applied a class-weighted logistic regression. Individuals in the RESH group were assigned a weight of 1, whereas those in the NRESH group were assigned a weight of 0.5. This weighting scheme ensured that both groups contributed comparably to the parameter estimation, thereby reducing potential bias arising from unequal sample sizes. The results remained consistent with our original analyses, indicating that the observed associations between amygdala–SMA connectivity and recurrent self-harm are robust and not driven by class imbalance (Table S11).

**Table S6 Logistic regression results for the association between SMA_L and outcome, weighted by propensity score weighting**

| **Variables** | **Bsaeline** | | **Change(T3-T1)** | |
| --- | --- | --- | --- | --- |
|  | OR (95%CI) | *P* | OR (95%CI) | *P* |
| Sex | 5.40(1.57, 18.38) | 0.007** | 3.42(1.02, 11.40) | 0.04* |
| Age | 0.79(0.62, 1.02) | 0.07 | 0.76(0.58, 0.99) | 0.05 |
| SMA.L | 0.03(0.002, 0.30) | 0.003** | 18.58(1.53, 225.24) | 0.02** |
| BSS_Q1 | 0.87(0.45, 1.66） | 0.67 | 1.39(0.67, 2.87) | 0.38 |
| BSS_Q3 | 1.98(1.03, 3.83) | 0.04* | 2.53(1.23, 5.22) | 0.01** |
| BSS_Q8 | 1.36(0.78, 2.37) | 0.28 | 0.76(0.48, 1.19) | 0.23 |
| BSS_Q13 | 0.84(0.51, 1.39) | 0.50 | 1.25(0.75, 2.06) | 0.39 |

*OR* adjusted odds ratio of the multivariable binary logistic regression, which set repeated self-harm(Yes vs. No) as the dependent variable, the FC value and clinical variables (age and sex) as the independent variables, *95%CI* 95% confidence interval,*SMA.L*, left supplementary motor area; *BSS_Q1，* Beck Scale for Suicidal Ideation items 1; *BSS_Q3，* Beck Scale for Suicidal Ideation items 3; *BSS_Q8，* Beck Scale for Suicidal Ideation items 8; *BSS_Q13，* Beck Scale for Suicidal Ideation items 13

*P < 0.05, **P < 0.01, ***P < 0.001

**Table S7 Class-weighted logistic regression**

| **Variables** | **Bsaeline** | | **Change(T3-T1)** | |
| --- | --- | --- | --- | --- |
|  | OR (95%CI) | *P* | OR (95%CI) | *P* |
| Sex | 4.15(1.46, 13.88) | 0.01* | 3.77(1.32, 12.64) | 0.02* |
| Age | 0.87(0.66, 1.14) | 0.31 | 0.87(0.66, 1.14) | 0.32 |
| SMA.L | 0.02(0.0007, 0.41) | 0.01* | 20.51(1.98, 262.91) | 0.01** |
| BSS_Q1 | 0.78(0.35, 1.66） | 0.52 | 1.35(0.62, 3.09) | 0.46 |
| BSS_Q3 | 1.87(0.88, 4.14) | 0.11 | 2.34(1.09, 5.38) | 0.03* |
| BSS_Q8 | 1.36(0.75, 2.52) | 0.31 | 0.78(0.47, 1.26) | 0.32 |
| BSS_Q13 | 1.02(0.57, 1.81) | 0.95 | 1.30(0.73, 2.40) | 0.38 |

*OR* adjusted odds ratio of the multivariable binary logistic regression, which set repeated self-harm(Yes vs. No) as the dependent variable, the FC value and clinical variables (age and sex) as the independent variables, *95%CI* 95% confidence interval,*SMA.L*, left supplementary motor area; *BSS_Q1，* Beck Scale for Suicidal Ideation items 1; *BSS_Q3，* Beck Scale for Suicidal Ideation items 3; *BSS_Q8，* Beck Scale for Suicidal Ideation items 8; *BSS_Q13，* Beck Scale for Suicidal Ideation items 13

*P < 0.05, **P < 0.01, ***P < 0.001

**Whole-Brain ROI PLSC Analysis in RESH**

We employed Partial Least Squares Correlation (PLSC) analysis to examine the multivariate associations within the RESH group between longitudinal changes in brain connectivity (T3–T1, Δ) and suicidal measures. To ensure unbiased feature identification, the brain matrix (X) included functional connectivity changes between the amygdala and all 90 cortical regions defined by the AAL atlas, while the behavioral matrix (Y) included all 19 items of the Beck Scale for Suicide Ideation (BSS). Both matrices were mean-centered and normalized. The significance of Latent Variables (LVs) capturing maximal shared variance was assessed using 1,000 permutations. The stability of contributing variables was evaluated via bootstrapping (1,000 iterations), using a reliability threshold of |z| > 1.96. Analyses were conducted in R (v4.1.2) using the TExPosition and data4PCCAR packages. The results indicated that the first latent variable (LV1) accounted for 56% of the covariance between brain features and suicidal symptoms. Specifically, the PLSC analysis identified significant associations between the left supplementary motor area (L-SMA) and BSS items 1, 3, 4, 5, 7, 8, 9, 10, 13, 14, 15, 17, and 19 (Figure S2).

**Nested cross-validation**

To rigorously evaluate the predictive generalizability of our logistic regression models and prevent overfitting, we implemented a Nested Cross-Validation (Nested CV) framework. The outcome variable was binary (SH recurrence: Yes vs. No), and predictors included brain imaging features (e.g., L-SMA), suicidal measures (BSS items 1, 3, 8, 13), age, and sex. The procedure consisted of an outer loop and an inner loop. In the **outer loop**, the dataset was split into 5 stratified folds, with one-fold held out as an independent test set for unbiased model evaluation. Within each training fold of the outer loop, a **5-fold inner cross-validation** was performed to optimize the hyperparameter for ridge-regularized logistic regression (using the *glmnet* package). Model performance was assessed on the held-out test folds of the outer loop using Receiver Operating Characteristic (ROC) curves; the Area Under the Curve (AUC), sensitivity, and specificity were calculated for each fold and averaged. The Nested CV results demonstrated that the **Longitudinal Model of Change** achieved a mean AUC of 70.5% (mean sensitivity: 79.9%; specificity: 59.7%), whereas the **Baseline Model** achieved a mean AUC of 69.4% (mean sensitivity: 65.3%; specificity: 80.5%) **(Figure S3)**.

**Fig. S1** Receiver operating characteristic curve to assess the predictive ability of baseline and longitudinal changes for the repeated self-harm behavior at 6 months after-discharge. The red curve was the baseline data; The blue curve was the longitudinal changes(T3-T1). 95% CI: 95% confidence interval; ROC: receiver operating characteristic; FC: functional connectivity; AUC: area under the receiver operating characteristic curve

**Fig. S2** Partial least squares correlation analysis of functional connectivity and clinical symptoms in RESH patients using all brain regions of interest.

**Fig. S3** Nested cross-validation ROC curves for the the Baseline Model and Longitudinal Change Model.

**Abbreviations**

| Abbreviations | Definitions |
| --- | --- |
| MD | Mood Disorder |
| BD | Bipolar Disorder |
| MDD | Major Depressive Disorder |
| SH | Self-harming |
| RESH | Repeated episodes of self-harm |
| NRESH | No-repeated episodes of self-harm |
| SA | Suicide Attempt |
| NSSI | Non-suicidal Self-injury |
| DSM-IV | The Diagnostic and Statistical Manual of Mental Disorders, fourth edition |
| K-SADS-PL | The Schedule for Affective Disorders and Schizophrenia for School-age Children Present and Lifetime Version |
| HAMD-17 | 17-item Hamilton Depression Rating Scale |
| HAMA | Hamilton Anxiety Rating Scale |
| BSS | Beck Scale for Suicide Ideation |
| FC | Functional connectivity |
| PLSC | Partial least squares correlation |
| ROC | Receiver Operating Characteristic |
| AUC | Area Under Curve |
| IFG | Inferior frontal gyrus |
| PrCG | Precentral gyrus |
| SFG | Medial superior frontal gyrus |
| ANG | Angular gyrus |
| MFG | Middle frontal gyrus |
| IPL | Inferior parietal lobule |
| SMA | Supplementary motor area |
| PHG | ParaHippocampal |
| IFGoperc | Inferior frontal gyrus, orbital part |
| ORBmid | Middle frontal gyrus, orbital part |
| LING | Lingual |
| CAL | Calcarine |
| STG | Superior temporal gyrus |
| SPG | Superior parietal lobule |
| MTG | Middle temporal gyrus |
| SOG | Superior occipital gyrus |
